# Inputs of Terrestrial Dissolved Organic Matter Enhance Bacterial Production and Methylmercury Formation in Oxic Coastal Water

**DOI:** 10.3389/fmicb.2022.809166

**Published:** 2022-07-27

**Authors:** Juanjo Rodríguez, Agneta Andersson, Erik Björn, Sari Timonen, Sonia Brugel, Aleksandra Skrobonja, Owen Rowe

**Affiliations:** ^1^Department of Ecology and Environmental Sciences, Umeå University, Umeå, Sweden; ^2^Department of Microbiology, University of Helsinki, Helsinki, Finland; ^3^Umeå Marine Research Centre (UMF), Umeå University, Hörnefors, Sweden; ^4^Department of Chemistry, Umeå University, Umeå, Sweden; ^5^ALS Scandinavia, Luleå, Sweden; ^6^Helsinki Commission (HELCOM), Baltic Marine Environment Protection Commission, Helsinki, Finland

**Keywords:** methylmercury, bacterial communities, terrestrial dissolved organic matter, shotgun metagenomics, climate change

## Abstract

Methylmercury (MeHg) is a potent neurotoxin commonly found in aquatic environments and primarily formed by microbial methylation of inorganic divalent mercury (Hg(II)) under anoxic conditions. Recent evidence, however, points to the production of MeHg also in oxic pelagic waters, but the magnitude and the drivers for this process remain unclear. Here, we performed a controlled experiment testing the hypothesis that inputs of terrestrial dissolved organic matter (tDOM) to coastal waters enhance MeHg formation *via* increased bacterial activity. Natural brackish seawater from a coastal area of the Baltic Sea was exposed to environmentally relevant levels of Hg(II) and additions of tDOM according to climate change scenarios. MeHg formation was observed to be coupled to elevated bacterial production rates, which, in turn, was linked to input levels of tDOM. The increased MeHg formation was, however, not coupled to any specific change in bacterial taxonomic composition nor to an increased abundance of known Hg(II) methylation genes. Instead, we found that the abundance of genes for the overall bacterial carbon metabolism was higher under increased tDOM additions. The findings of this study may have important ecological implications in a changing global climate by pointing to the risk of increased exposure of MeHg to pelagic biota.

## Introduction

Mercury (Hg) has been ranked as one of the most hazardous contaminants in biota, playing a major role in the overall failure of good environmental status in aquatic systems when considering hazardous substances (i.e., the integrated contamination status, HELCOM, 2018). Toxicity is largely associated with methylmercury (MeHg), a potent neurotoxin that can be bioaccumulated and biomagnified in food webs (Boening, 2000; Ullrich et al., 2001). The formation of MeHg in nature occurs through methylation of Hg(II) and is predominantly performed by microorganisms carrying the *hgcAB* gene pair (Gilmour et al., 2013; Parks et al., 2013; Podar et al., 2015). Numerous studies have described Hg methylation in anoxic sediments, where sulfate-reducing bacteria, iron-reducing bacteria, and methanogens are commonly detected as the main methylators in these anaerobic environments (Gilmour et al., 2013; Podar et al., 2015; Paranjape and Hall, 2017). However, while anoxic sediments are the primary source of MeHg formation, a number of studies have reported environmentally high concentrations of MeHg in pelagic environments, and Hg methylation has been detected in surface and intermediate layers of the water column from different aquatic systems (Lehnherr et al., 2011; Kirk et al., 2012; Paranjape and Hall, 2017). Such observations have led to the hypothesis that significant biotic methylation of Hg may also take place in pelagic waters under oxic conditions, but the exact mechanism remains largely unknown as the gene pair *hgcAB*, as well as the bacterial species carrying them, are rarely found in oxic waters (Malcolm et al., 2010; Podar et al., 2015; Gionfriddo et al., 2016; Villar et al., 2020). Furthermore, it is still unclear whether *hgcAB* genes are simply induced by elevated concentrations of Hg(II) (Goñi-Urriza et al., 2015). While weak correlations have been found between *hgcA* at increasing Hg levels, organic matter has shown to have the strongest effects on the abundance of *hgcA* in natural communities (Liu et al., 2014). These observations have led to the hypothesis that Hg methylation, rather, is linked to the overall activity level of bacterial metabolism (Regnell and Watras, 2018).

Dissolved organic matter (DOM) and dissolved organic carbon (DOC) have been shown to promote methylation of Hg in different manners, for example by stimulating microbial activity and thus methylation processes (Hall et al., 2004; Bravo et al., 2017; Herrero Ortega et al., 2018), by providing methyl groups for methylation (Paranjape and Hall, 2017), or by enhancing the solubility of HgS(s) mineral phases (cinnabar; Waples et al., 2005). A number of studies have reported a direct relation between DOM concentration/composition, bacterial activity, and MeHg formation in various aquatic environments (Bravo et al., 2017; Herrero Ortega et al., 2018), including boreal lakes (Braaten et al., 2014), lagoons (Faganeli et al., 2012), wetlands (Hall et al., 2008), open marine waters (Cossa et al., 2009; Sunderland et al., 2009), and sediments (Mazrui et al., 2016). The Baltic Sea is predicted to undergo higher inputs of DOM from terrestrial runoff and river inflow as a result of increased precipitation regimes in the Northern hemisphere (Meier et al., 2011; Andersson et al., 2015). Increased DOM inputs may lead to higher inputs of Hg and MeHg (Krabbenhoft and Sunderland, 2013) and may promote enhanced MeHg formation in the aquatic ecosystem.

Recent studies have observed bell-shaped relationships between Hg bioavailability, MeHg bioaccumulation, and concentration of DOM and DOC, where bioavailability and bioaccumulation are enhanced with increasing DOM/DOC concentrations up to a threshold (~8.5 mg C L^−1^), after which an inverted trend is observed (Chiasson-Gould et al., 2014; French et al., 2014). Furthermore, Hg methylation has been suggested to take place predominantly within a few hours (~24 h) after entering aquatic ecosystems, and to a greater extent under non-equilibrium conditions, that is, before inorganic Hg becomes complexed with different molecules present in the DOM pool (Chiasson-Gould et al., 2014). This may result in a rapid methylation of Hg(II) as it enters the aquatic systems *via* atmospheric deposition (Hissler and Probst, 2006; Mason et al., 2012; Chiasson-Gould et al., 2014).

This study examined the taxonomic and functional composition of natural bacterioplankton communities from a coastal area of the Bothnian Sea. A microcosm experiment was conducted where natural microbial communities were exposed to environmentally relevant levels of Hg(II) and increasing concentrations of terrestrial dissolved organic matter (tDOM) according to future climate change scenarios. We addressed three main hypotheses currently under debate related to Hg methylation: (1) MeHg is formed from Hg(II) in oxic waters by natural microbial communities in the absence or low abundance of the *hgcAB* gene pair; (2) MeHg formation is coupled to the overall bacterial biochemical activity, which is enhanced by the addition of tDOM; and (3) A rapid response of bacterial biochemical activity leads to MeHg formation within few hours after the input of Hg(II) to oxic DOM-rich waters.

## Materials and Methods

### Experimental Design and Treatments

The experiment was carried out in September 2017 over a period of 12 days at Umeå Marine Sciences Center (UMF), Sweden. On September 11th, 12 aquaria were filled with 37 l of GF/F filtered (Whatman^®^, 0.7 μm pore size for the isolation of the bacterial fraction) seawater (salinity of 3) collected from the research station central supply of seawater, whose intake is placed 1 km offshore of the UMF facility (63°34′N, 19°54′E). The aquaria were placed in a climate room at a constant temperature of 15°C and light intensity of ~26 μE m^−2^ s^−1^. Ambient air was gently pumped into the aquaria to ensure a constant aeration of the seawater. The four experimental treatments, in triplicate, consisted of natural seawater exposed to: (1) ambient conditions (Control), i.e., natural DOC concentration and no Hg addition; (2) addition of Hg (Hg^+^); (3) addition of DOC corresponding to ~40% increase from natural levels, as well as addition of Hg (DOC_40_-Hg^+^); and (4) addition of DOC corresponding to ~70% increase from natural levels, plus addition of Hg (DOC_70_-Hg^+^).

The increase of DOC concentration was achieved by the addition of a terrestrial dissolved organic matter (tDOM) extract derived from humic-rich soil material collected locally at the Öre estuary (63° 59.5469′N, 19° 80.9809′E) from the top 5–10 cm of the soil profile. After collection, the soil was homogenized, sieved through 4–6 mm sieve nets, and mixed with a chelating resin (Diaion^®^ CR11, sodium form, Sigma Aldrich) and MQ water (18.2 MΩ cm^−1^, at 25°C and 5 ppb TOC). Mechanical pumping was applied for vigorous stirring and the homogenate was centrifuged (10,000 rpm, 10 min, rotor JA-14). A serial filtration was performed down to 0.2 μm in order to obtain a soil extract where cellular organisms were excluded. The DOC concentration of the extract was analyzed (see below) to determine the volume of required addition to achieve the treatments (Table 1). The tDOM extract was added on September 11th, ~6 h after the aquaria were filled.

**Table 1:** Experimental layout showing concentrations of dissolved organic carbon (DOC) and total mercury (Hg) in the different treatments achieved after the corresponding additions.

Treatment	Replicates	DOC (mg L^−1^)	Hg (pM)	Temp. (°C)
CONTROL	3	4.05 (±0.02)	0	15
Hg^+^	3	4.05 (±0.03)	∼250	15
DOC_40_-Hg^+^	3	5.59 (±0.03)	∼250	15
DOC_70_-Hg^+^	3	6.81 (±0.05)	∼250	15

Hg(II) was added as a ~3 μM HgCl_2_ working solution prepared by serial dilutions with Milli-Q water from a 0.3 mM stock solution in 0.1 M HCl. On September 13th, after ~48 h from the tDOM addition, the HgCl_2_ working solution was added into the corresponding aquaria to reach a final concentration of ~250 pM Hg(II) in treatments 2–4.

Samples were collected five times over the duration of the experiment: ~12 h after the addition of the tDOM extract (referred as Day 1 from the start of the experiment), 13 h and 27 h after the addition of Hg (referred as day 3 and day 4, respectively), and after 8 and 12 days from the outset of the experiment (referred as day 8 and day 12, respectively).

### Total Hg and MeHg Analyses

Total Hg was determined following US EPA Method 1631E combined with isotope dilution analysis. Briefly, samples were preserved with 0.1 M HCl immediately after collection. Prior to analysis, samples were digested according to US EPA Method 1631E using 1% BrCl for 24 h, after which NH_2_OH.HCl was added for BrCl neutralization. A ^200^Hg isotope enriched standard was added for isotope dilution analysis. Thereafter, analyses were run by using an on-line cold vapor generation system (HGX-200, Cetac) with SnCl2 reduction connected to a PerkinElmer/Sciex ELAN DRCe inductively coupled plasma mass spectrometer. Methylmercury was determined as previously described (Lambertsson and Björn, 2004; Munson et al., 2014). Briefly, samples were acidified to 0.1 M HCl immediately after collection. Prior to analysis, a Me^200^Hg isotope enriched standard was added and a combination of 10 M sodium hydroxide (NaOH), 200 μl of 2 M acetate buffer, and 300 μl of 2.5% ascorbic acid solution was used to adjust the pH of the samples to 4.8. Ethylation of MeHg species was achieved by the addition of sodium tetraethylborate (STEB), after which samples were purged and trapped onto Tenax adsorbent matrix. Thermal desorption-gas chromatography combined with inductively coupled plasma mass spectrometry (TDGC-ICPMS, 6890 Agilent GC, and 7700 ICPMS) was employed for thermal desorption of ethylated mercury species. For quality control, lobster hepatopancreas (TORT-3, National Research Council Canada; certified MeHg concentration of 0.137 ± 0.012 mg kg^−1^) was determined by TDGC-ICPMS after extraction according to Qvarnström and Frech (2002), with an average recovery of 99.8 ± 5.7% (*n* = 9). The concentrations of enriched Hg isotope standards were regularly determined by reversed isotope dilution analysis. All measured ICPMS isotope ratios were mass bias corrected using a linear mass bias model with a correction factor of 0.994. Method blanks consisting of MQ water (>18 MΩ, Merck Millipore Advantage A10 Ultrapure) and all reagents for total Hg or MeHg samples were regularly analyzed and subtracted from all sample concentrations. In addition, the content of total Hg and MeHg in the tDOM soil extract was analyzed and subsequently subtracted from the values measured in the seawater where the extract was added (750 ng g^−1^C of total Hg, and 2.68 ng g^−1^C of MeHg).

### Nutrients DOC

Samples for DOC, total dissolved phosphorus (TDP), and total dissolved nitrogen (TDN) were filtered through 0.2 μm filters (Supor Membrane Syringe Filter, non-pyrogenic; Acrodisc^®^). Total dissolved phosphorus and TDN were determined using a Seal QuAAtro39 autoanalyzer after an oxidation step using peroxodisulfate (Grasshoff et al., 1983). Dissolved organic carbon samples were acidified with 375 μl of 1.2 M HCl and stored at 4°C before analysis. Dissolved organic carbon was measured by the high-temperature catalytic oxidation method using a Shimadzu TOC-5000 instrument (Shimadzu Corporation) with platinum-coated Al_2_O_3_ granulates as a catalyst.

### Bacterial Production

Samples for chlorophyll-a (Chl-a) were filtered onto Whatman^®^ GF/F filters, extracted overnight in the dark in 95% ethanol, and then measured on a PerkinElmer LS 30 spectrofluorometer (Waltham, MA, United States) operating at excitation and emission wavelengths of 433 and 673 nm, respectively.

Bacterial net production (BP) was measured using the [^3^H-methyl]-thymidine incorporation method (Fuhrman and Azam, 1982). Three 1 ml replicates and one killed control were incubated in the dark at 15°C for 1 h with [^3^H-methyl]-thymidine (84 Ci mmol^−1^, PerkinElmer^®^, Massachusetts, United States) at a final concentration of 24 nM. The incubation was stopped by the addition of 100 μl of ice-cold 50% trichloroacetic acid (TCA) and the samples were then centrifuged at 4°C at 13,000 rpm for 10 min. The resulting pellet was washed with 5% trichloroacetic acid (TCA) and, and after adding 1 ml of scintillation cocktail (OptiPhase 3, PerkinElmer), the samples were analyzed in a scintillation counter (PerkinElmer Tri-Carb 2910 TR). Bacterial production was calculated using a conversion factor of 1.4 × 10^18^ cells mol^−1^ (Wikner and Hagström, 1999) and a carbon conversion factor of 20 fg C cell^−1^ (Lee and Fuhrman, 1987).

### DNA Extraction and Shotgun Metagenomics

Water samples for DNA extraction were collected at days 3, 4, 8, and 12, and immediately fixed by adding a phenol–ethanol stop solution (5% phenol, 95% absolute ethanol) in a 1:10 dilution. The fixed samples were then filtered (0.6–1 L) onto 0.22 μm 47 mm PVDF membrane filters (Durapore^®^) and immediately stored at −80°C until further processing. Genomic DNA was extracted using an AllPrep DNA/RNA/Protein Mini Kit (Qiagen^®^) according to the manufacturer’s protocol. These samples were submitted to Science for Life Laboratory,^1^ Sweden, for library preparation and subsequent 2 × 150 pair-end shotgun sequencing on an Illumina^®^ NovaSeq 6000 platform.

### Bioinformatics Analysis

The FASTQ files generated by Illumina^®^ NovaSeq 6000 were processed using an adaptation of the META-pipe analysis pipeline developed by ELIXIRNorway (Robertsen et al., 2017), where *FastQC* was used for quality control, *SeqPrep* for merging the overlapping reads, *Trimmomatic* for trimming adapters and filtering out reads with average quality below phred score 20, and *Megahit* for assembling reads into contigs. For taxonomic profiling, *predict_16s.py* was used to predict/find rRNA sequences. *Megablast* (*blastn*) was run against Silvamod database (based on Silva rRNA database), after which *LCAClassifier* was employed to produce a taxonomic classification at a 97% similarity threshold. Functional gene annotation was carried out using Megan6 Community Edition (Megan 6.15.1; Huson et al., 2016). The contigs previously assembled by *Megahit* were blasted against an NCBI non-redundant database created using DIAMOND with options *-F 15 –range-culling –top 10* for long reads. Using the program *Meganizer* in the long-read mode (*–longReads*), the resulting Diamond alignment archives (DAA) were mapped against the InterPro2GO protein families database (Mitchell et al., 2014) for functional classification of the reads by Gene Ontology assignment based on the metagenomic GO-slim (Hunter et al., 2013). The meganized files were imported into InterPro2Go viewer for visualization and subsequent analyses. Sequences have been deposited with links to BioProject accession number PRJNA777782 in the NCBI BioProject database.^2^

### Statistical Analyses

Statistical analyses were performed in the R project environment (v. 3.5.2) unless specified otherwise. Significant differences between treatments and sampling days from the experimental factors considered in this study (i.e., DOC, nutrients, Hg, MeHg, Shannon index, bacterial production, and abundance of bacterial taxa and functional genes) were analyzed by repeated-measures ANOVA, followed by Tukey’s Honest Significant Differences test with pairwise comparisons. For analyses of bacterial community structure and abundance of functional genes, raw read counts were normalized by down-sampling to the smallest library size. To estimate the alpha diversity of bacterial communities, Shannon’s diversity index was determined using the R function *diversity* from *vegan* package (v. 2.5.6). Bacterial community structure dissimilarities between treatments were determined and visualized by Principal Component Analysis (PCA) using the standard R function *prcomp*. The R package *phyloseq* (v. 1.26.1) was used to calculate the relative abundance of bacterial taxa present in the communities. Differentially abundant functional genes were presented in heatmaps (*heatmaply* R function, package *heatmaply* v.0.16.0) based on log2 fold-change compared to the unexposed control. Raw read counts were normalized by randomly down-sampling to the smallest library size (rarefaction; *phyloseq* v. 1.26.1).

## Results

### Dissolved Organic Carbon and Nutrients

**Figure 1:**
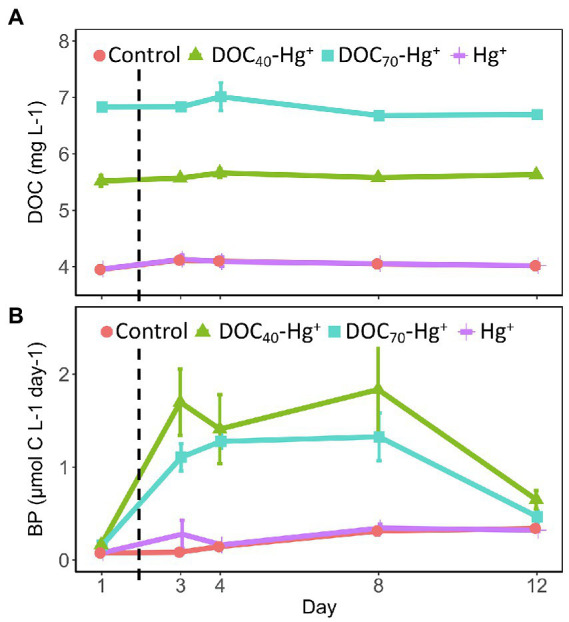
Concentration of dissolved organic carbon **(A)** and bacterial production **(B)** in the different treatments throughout the experiment. The dashed line indicates the addition of Hg(II). Error bars represent the standard error (*n* = 3).

The addition of the tDOM extract resulted in increased DOC concentrations representative of the different treatments (Figure 1A). Thus, the unexposed control and the Hg^+^ treatment showed an average DOC concentration equivalent to natural levels (~4.0 mg L^−1^ ± 0.02, *n* = 15; Andersson et al., 2018). On the other hand, DOC_40_-Hg^+^ showed an average DOC concentration of 5.6 mg L^−1^ (±0.03, *n* = 15), which corresponds to a ~40% increase from natural levels, while the average DOC concentration in DOC_70_-Hg^+^ was 6.8 mg L^−1^ (±0.05, *n* = 15), representing a ~70% increase from natural levels. The DOC concentration remained stable over the course of the experiment in all treatments.

The addition of tDOM extract also led to increasing concentrations of TDP and TDN corresponding to the amount of extract added, with the unexposed control and Hg^+^ showing natural levels (5.7 μg L^−1^ ± 0.29 TDP, *n* = 30; 182.8 μg L^−1^ ± 2.2 TDN), and tDOM treatments (i.e., DOC_40_-Hg^+^ and DOC_70_-Hg^+^) showing increasing concentrations, respectively (Supplementary Figures S1A,B). Total dissolved phosphorus concentrations exhibited similar trends in all treatments, with decreasing concentrations until day 4 followed by increases toward the end of the experiment. Total dissolved nitrogen also showed similar trends in all treatments, with slightly lower concentrations on day 4 and higher concentrations by the end of the experiment compared to the initial values at day 1.

### Total Mercury and Methylmercury

HgT concentration remained low over the experiment in the unexposed control (11.5 ± 1.66 pM, *n* = 15; Supplementary Figure S1C). Addition of HgCl_2_ led to an increase of the HgT concentration (174–289 pM), which was markedly higher than in the control (repeated-measures ANOVA value of *p*s ≤ 0.001), but not significantly different between the treatments receiving HgCl_2_ (i.e., Hg^+^, DOC_40_-Hg^+^ and DOC_70_-Hg^+^; repeated-measures ANOVA value of *p*s > 0.05). Due to high variability of HgT samples from days 1 and 3, these two time points were not further evaluated with respect to HgT (raw values are shown in Supplementary Table S1). While HgT concentration slightly decreased in Hg^+^ and DOC_40_-Hg^+^ by day 12, concentrations in treatment DOC_70_-Hg^+^ showed a marked increase, becoming significantly higher than the concentration found in Hg^+^ (TukeyHSD adj. value of *p* = 0.005).

**Figure 2:**
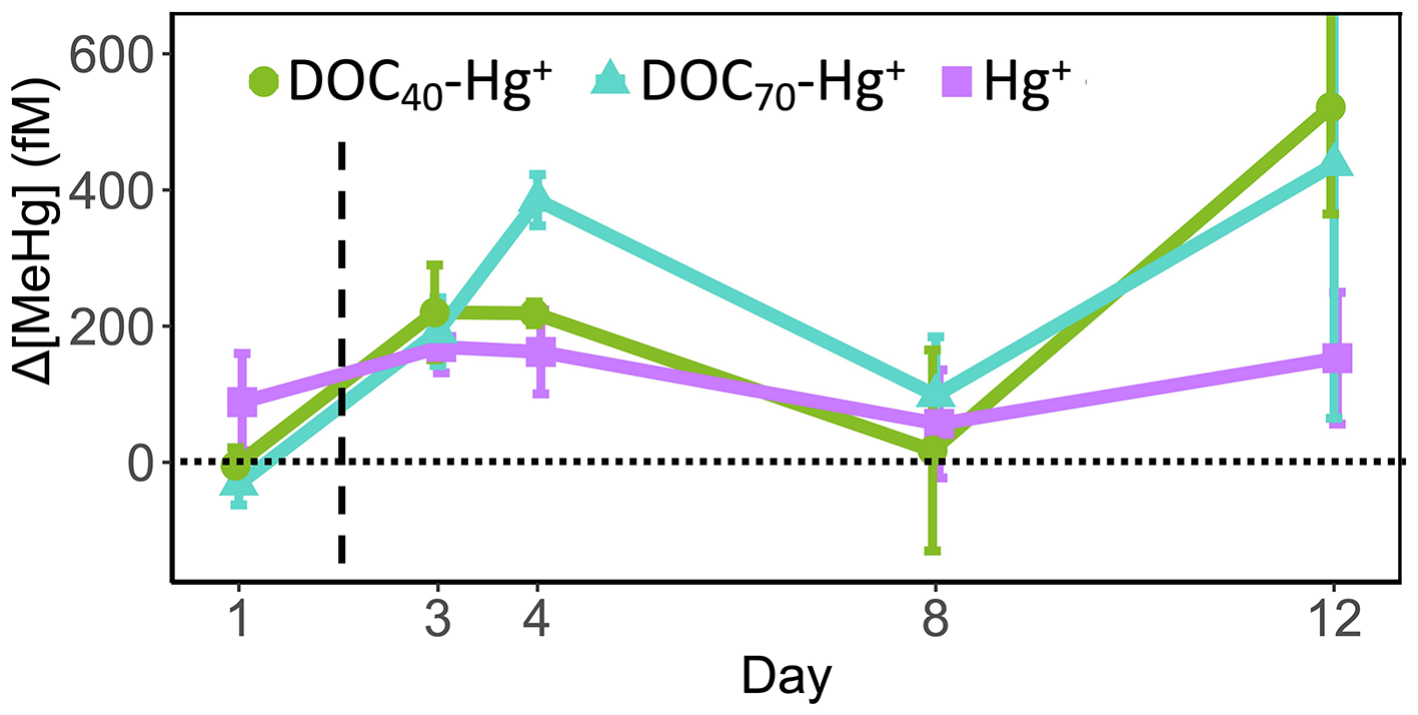
Increment of methylmercury (ΔMeHg) concentration over the unexposed control throughout the experiments in the treatments with addition of Hg(II). Δ[MeHg] = Value_(treatment)_ − Value_(Control)_. The dashed line indicates the addition of Hg(II). Error bars represent the standard error (*n* = 3).

The concentration of MeHg was normalized against the control to visualize the treatment effects, and was observed to increase in all Hg treatments compared to the unexposed control (Figure 2; Supplementary Figure S1D). Prior to the addition of HgCl_2_ (i.e., at day 1), MeHg concentration was not significantly different between any of the treatments, ranging between 116 and 236 fM. The addition of HgCl_2_ quickly led to a significant increase in MeHg throughout the experiment, especially in the treatments with the addition of tDOM (Figure 2; Supplementary Figure S1D). By day 3, the MeHg concentration increased significantly in all Hg treatments compared to the control (Δ169–Δ220 fM), with no significant differences between these three treatments. The MeHg concentration continued increasing in DOC_70_-Hg^+^ by day 4 (Δ385 ± 38 fM, *n* = 3), showing significant differences compared to the other Hg treatments (TukeyHSD adj. value of *p*s < 0.05). These differences between Hg treatments and the unexposed control were reduced by day 8 and became greater again by day 12, where DOC_40_-Hg^+^ showed significantly higher MeHg concentration compared to the control (Δ522 ± 157 fM, *n* = 3, TukeyHSD adj. *p*-value = 0.03). It is worth noting that the uncorrected concentration of MeHg was found to increase in all treatments by days 8 and 12, including the control (Supplementary Figure S1D). Considering the low concentration of total Hg in the unexposed control (Supplementary Figure S1C), the magnitude of increment in MeHg concentration, and the large standard error values at these sampling days, we regard contamination during sampling and chemical analyses as a plausible explanation for the high values of MeHg observed in the unexposed control at days 8 and 12.

### Bacterial Production and Links to MeHg Formation

Bacterial production was similar in all treatments at the start of the experiment. After 3 days of incubation, the production increased up to 10-fold in the treatments with tDOM addition (i.e., DOC_40_-Hg^+^ and DOC_70_-Hg^+^) compared to the control and the Hg^+^ treatment (TukeyHSD adj. *p*-values < 0.05, Figure 1B). From day 8 onward, bacterial production decreased in the tDOM treatments, although the values remained slightly above the original levels at day 1. In the control and Hg^+^, bacterial production was relatively stable all through the experiment, although showing a small gradual increase that led to significantly higher bacterial production by day 12 compared to the initial values at day 1 (TukeyHSD adj. *p*-values < 0.01). The highest values of bacterial production observed in the tDOM treatments (2–3 μmol C × L^−1^ × day^−1^) were in the range of those previously reported in coastal waters from nearby areas (Andersson et al., 2018).

**Figure 3:**
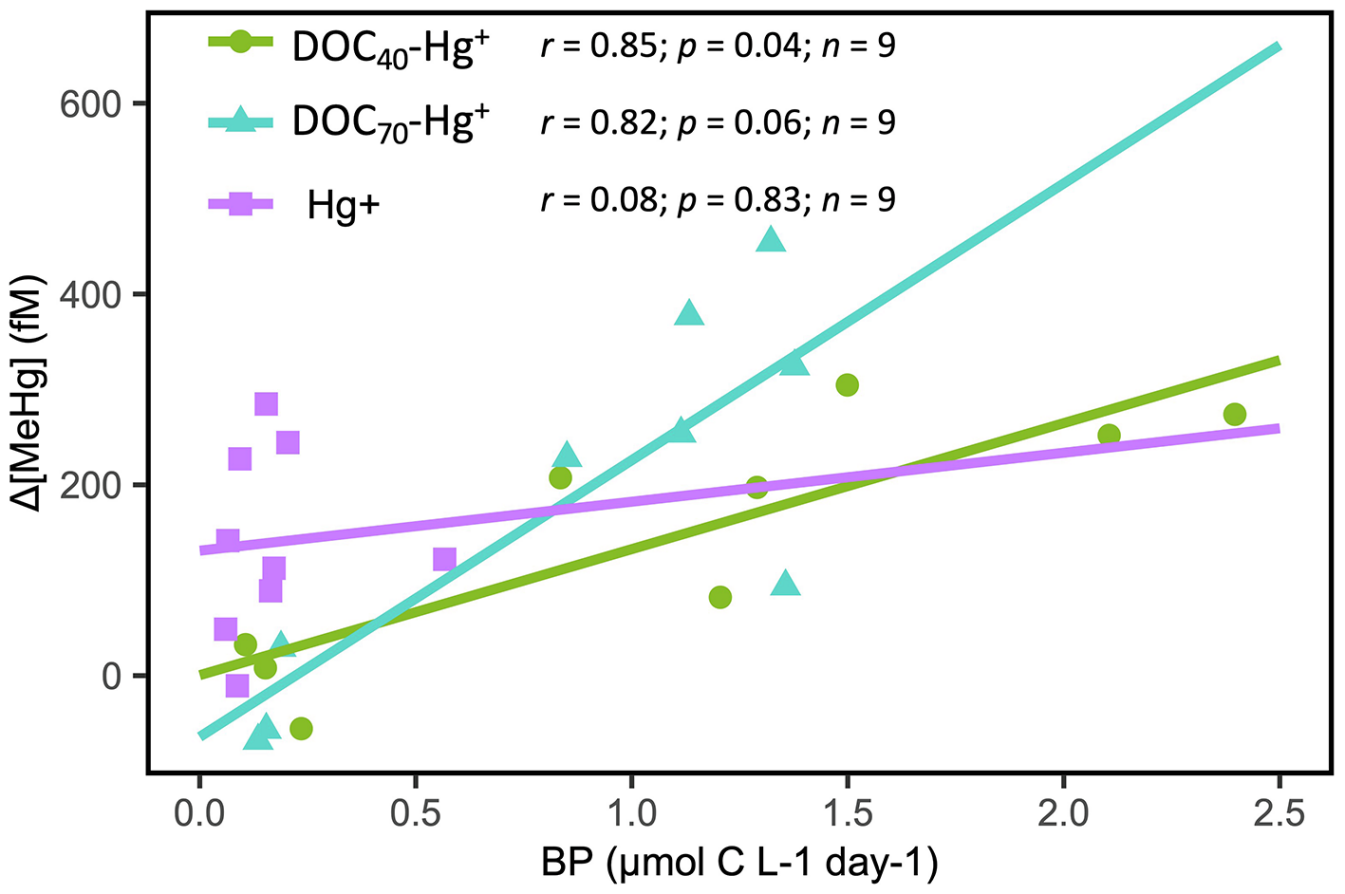
Pearson correlation between MeHg concentration (normalized against control; Δ[MeHg]) and bacterial production (BP) over the first 4 days of the experiment in the DOM and Hg treatments. Pearson correlation coefficients (*r*), *p*-values (*p*) and number of observations (*n*) are shown.

A significant positive correlation between bacterial production and MeHg concentration was observed during the first 4 days of the experiment in DOC_40_-Hg^+^ and DOC_70_-Hg^+^ (Figure 3), while such a relationship could not be found in the control and Hg^+^ treatment.

### Bacterial Diversity and Community Structure

After quality control, merging and trimming, a total of 606,745,688 sequences (13,190,124 ± 4,967,653 sequences per sample) were assembled into 15,504,957 contigs (337,064 ± 86,765 per sample) ranging between 200 and 303,367 bp (791 bp in average). Among them, 1,049,295 sequences were assigned to 16S rRNA gene, upon which taxonomical and biodiversity analyses were performed.

**Figure 4:**
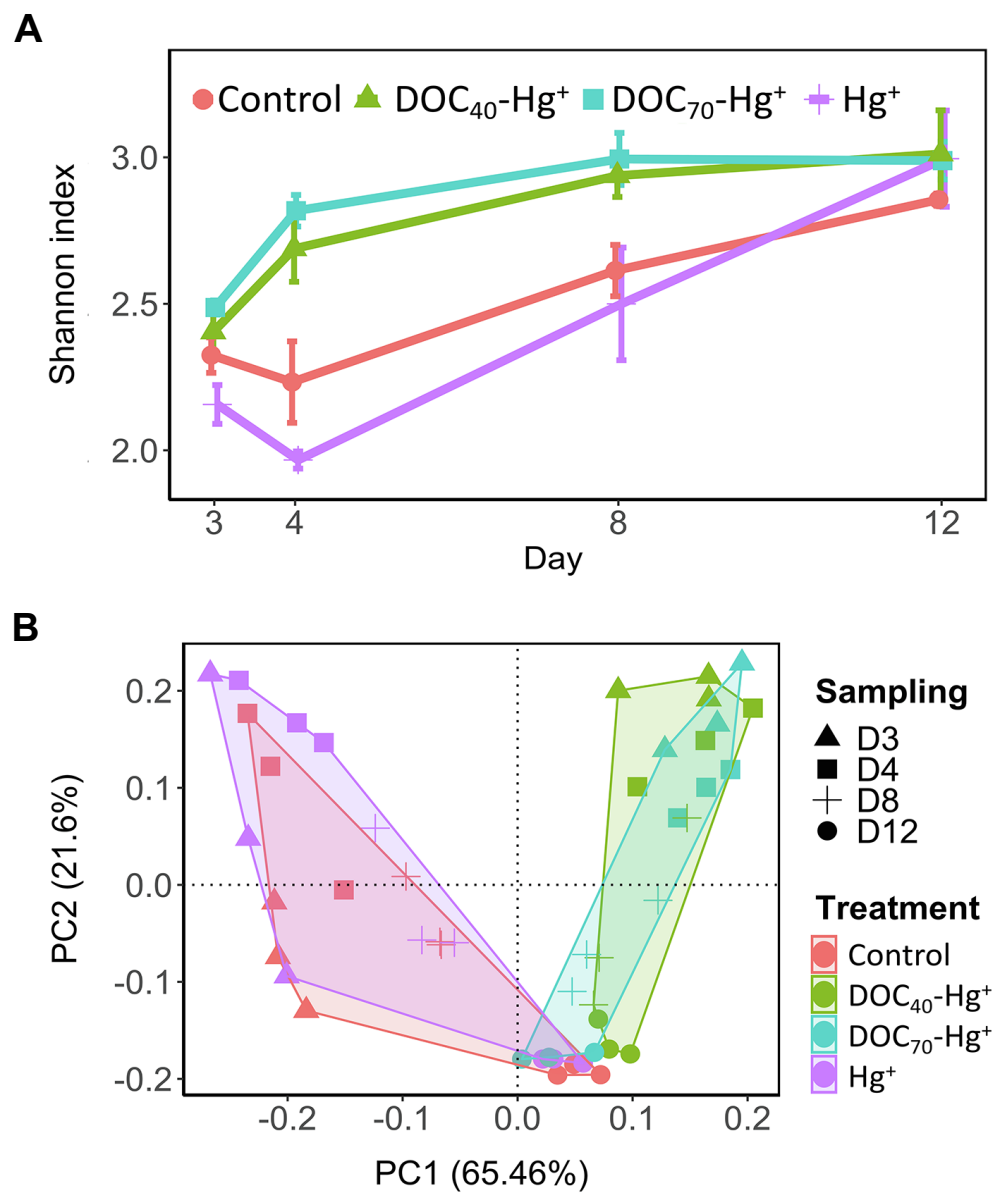
Diversity (Shannon’s index at genus level) **(A)** and community structure **(B)** of bacterial communities from the different treatments over the experiment. Error bar represent the standard error (*n* = 3).

**Figure 5:**
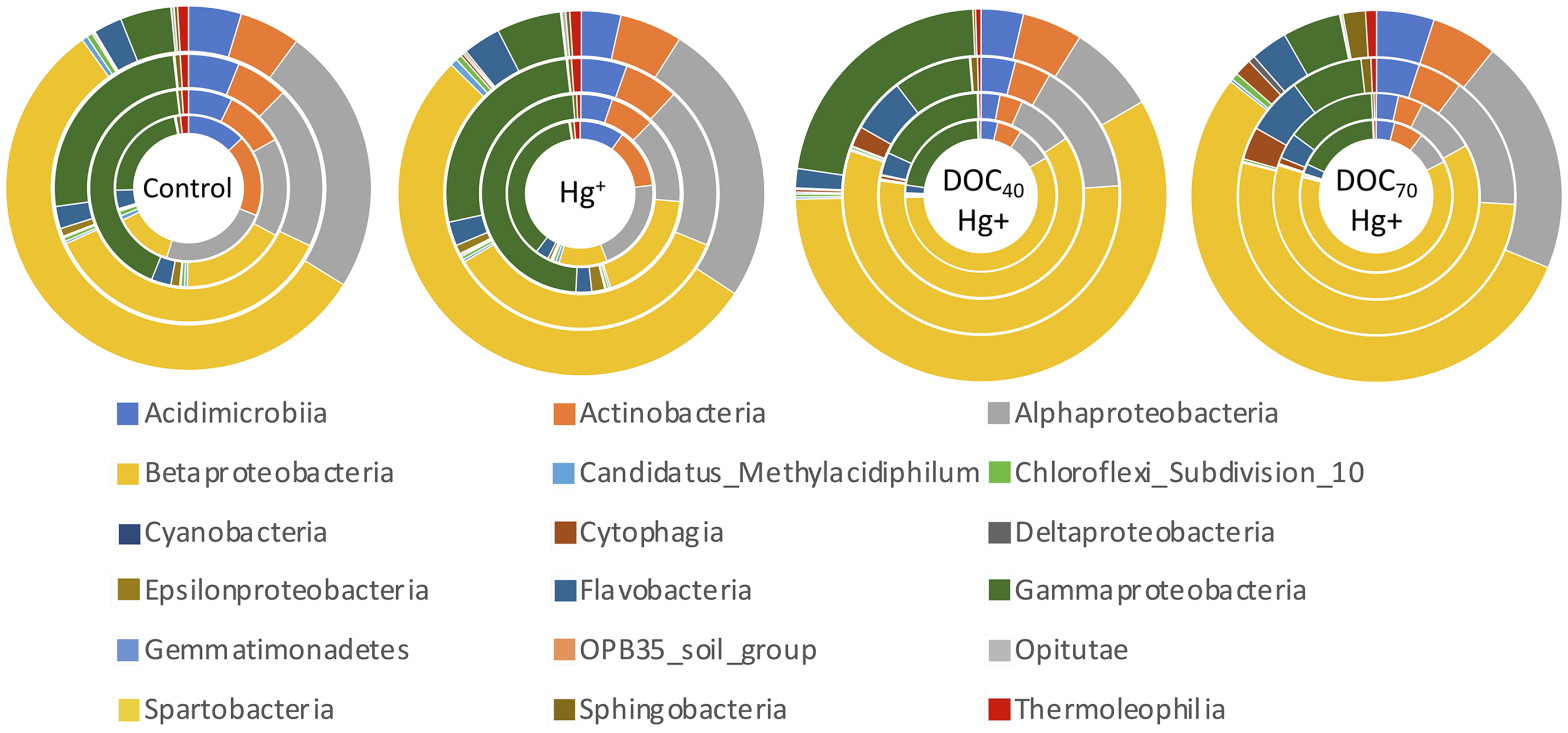
Relative abundance (%) of bacterial classes. Sampling days are represented by rings ordered from inside out: day 3–day 12.

Overall, bacterial diversity (Shannon index) at the genus level increased in all treatments over the course of the experiment (Figure 4A). Treatments with tDOM addition showed significantly higher diversity values at days 4 and 8 compared to the control and Hg^+^ (TukeyHSD adj. value of *p*s < 0.007), while these latter two did not differ significantly. However, all treatments reached similar diversity values by the end of the experiment (day 12). Similarly, a Principal Component Analysis revealed differences in community structure at early stages of the experiment between treatments with tDOM addition and those without (i.e., the unexposed control and Hg^+^ treatment), after which all treatments showed increasingly similar community structures over the course of the experiment (Figure 4B). Furthermore, similar trends were observed in the taxonomic composition at the class level (Figure 5), where some of the most abundant bacterial classes showed conspicuous differences in relative abundance between tDOM and non-tDOM treatments during the first stages of the experiment while reaching increasingly similar relative abundances toward the end of the experiment (TukeyHSD adj. value of *p*s > 0.05 at day 12). For example, Betaproteobacteria represented <15% in the non-tDOM treatments at the beginning of the experiment, and, yet, this class was found to represent as much as ~50% of the relative abundance in both tDOM and non-tDOM treatments on day 12.

At lower taxonomic levels, similar trends were observed for the abundant taxa. For example, bacterial taxa such as families Acidimicrobiaceae, Chesapeake-Delaware-Bay, Methylophilaceae, and Sporichthyaceae, and genera CL500-29 marine group and HgCl-clade, were significantly more abundant in non-tDOM treatment during the first part of the experiment while showing similar relative abundances as the experiment progressed (Supplementary Figures S2D,E). On the other hand, the family Oxalobacteraceae and the genus *Janthinobacterium* are examples of taxa with remarkably higher abundances in tDOM treatments (TukeyHSD adj. *p*-value < 0.05), although these differences were more pronounced during the first days (Supplementary Figures S2D,E).

### Functional Gene Abundances Under the Load of Hg(II) and Increasing tDOM Concentration

**Figure 6:**
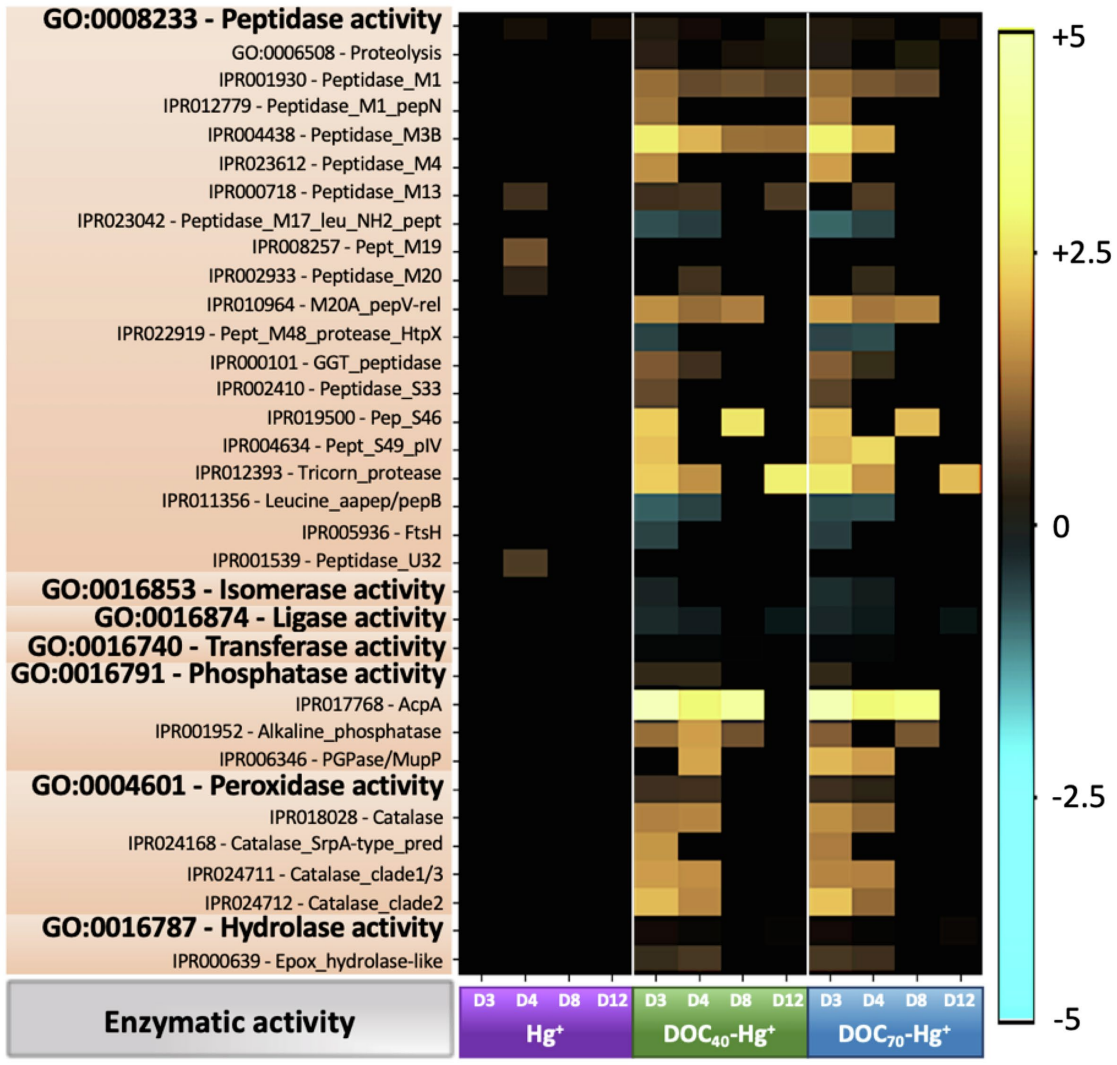
Heatmap showing Log2 fold-change abundance of functional genes involved in different enzymatic activities from treatments Hg^+^, DOC_40_-Hg^+^, and DOC_70_-Hg^+^ compared to the unexposed control. Warm colors (yellow) indicate overrepresentation (i.e., higher abundances), colder colors indicate underrepresentation, and black indicates non-significance (*p* > 0.05).

**Figure 7:**
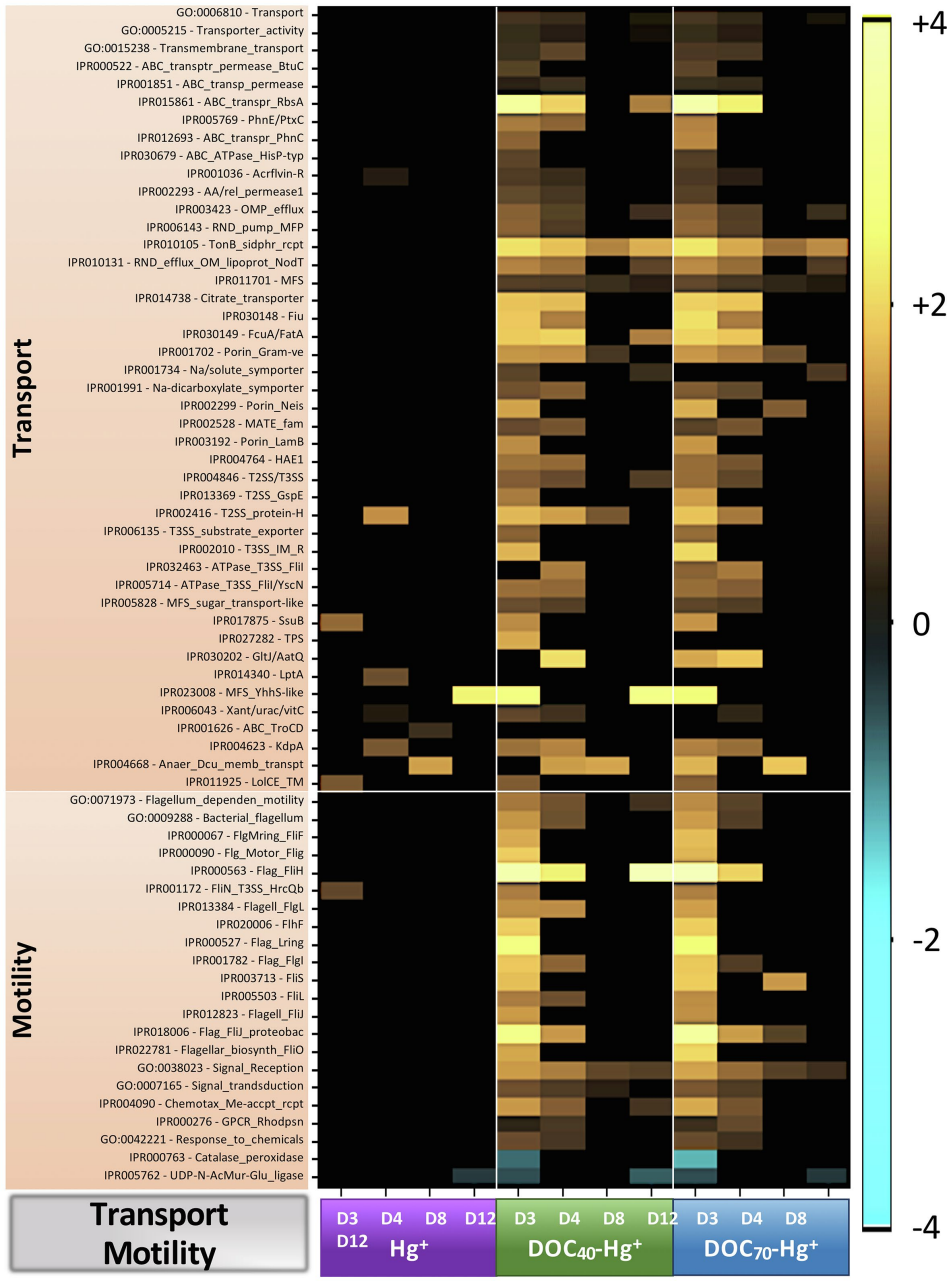
Heatmap showing Log2 fold-change abundance of functional genes involved in transport of molecules and cell motility from treatments Hg^+^, DOC_40_-Hg^+^, and DOC_70_-Hg^+^ compared to the unexposed control. Warm colors (yellow) indicate overrepresentation (i.e., higher abundances), colder colors indicate underrepresentation, and black indicates non-significance (*p* > 0.05).

A total of 6,958 functional genes (i.e., non-rRNA genes) were annotated and assigned to different gene ontology (GO) categories (InterPro database). Genes with <5 reads assigned across all treatments were discarded from further analyses. Statistical comparisons of abundance between the different treatments and the unexposed control resulted in the identification of 243 differentially represented genes (DRG), which were classified into broader functional categories such as metabolic processes (e.g., metabolism of DNA, nitrogen and iron/sulfur, generation of energy, etc.), transport, cell motility, enzymatic activity (e.g., peptidase, transferase, hydrolase, ligase or isomerase activity), and role as cellular components (structural constituents of ribosomes, membrane, cytoplasm, intracellular and extracellular compartments, etc.; Figures 6, 7; Supplementary Figure S3).

A total of 74 DRG were identified in bacterial communities from treatment Hg^+^ compared to the unexposed control. These differences were observed to occur within the first 27 h after the addition of Hg(II) (i.e., days 3 and 4) in 71.6% of the DRG, while only 28.4% of these genes were differentially abundant by days 8 and 12. In addition, 50.6% of these DRG were underrepresented and 49.4% were overrepresented compared to the control. On the other hand, up to 185 DRG were found in bacterial communities from tDOM treatments compared to the unexposed control, among which 44.9% were underrepresented and 55.1% overrepresented. These significant differences were exclusively observed up to 27 h after Hg(II) addition in up to 77.8% of these genes. Only 23% of all DRG showed significant differences between the three Hg^+^ treatments. A comprehensive overview of the differential abundance of these functional genes is provided in Appendix 1. Some noteworthy examples are presented below.

#### Genes Involved in Enzymatic Activity

Sequences assigned to genes involved in peptidase activity (GO:0008233) and proteolysis (GO:0006508) were overall slightly overrepresented in all Hg treatments, with a number of genes encoding for peptidases (e.g., *Peptidase_M1*, *Peptidase_M3B*, *Tricorn_protease*, *Pept_S49_pIV*, etc.) showing significantly higher abundances at different time points compared to the control (up to 6.5 fold-change, TukeyHSD adj. value of *p*s < 0.05; Figure 6). Genes involved in isomerase (GO:0016853), ligase (GO:0016874), and transferase (GO:0016740) activities were overall at significantly lower abundances in the tDOM treatments, particularly during days 3 and 4 (up to 1.22 fold-change, TukeyHSD adj. value of *p*s < 0.04). In addition, sequences related to phosphatase (GO:0016791), peroxidase (GO:0004601), and hydrolase (GO:0016787) activities were overrepresented particularly at days 3 and 4 (up to 1.4 fold-change, TukeyHSD adj. *p*-values < 0.035).

#### Genes Involved in Transport

Sequences assigned to genes involved in transport processes (GO:0006810; that is, directed movement of substances or cellular components into or out of a cell, or between cells), transporter activity (GO:0005215), and transmembrane transport (GO:0015238) were overall overrepresented in both tDOM treatments (up to 1.5 fold-change, TukeyHSD adj. *p*-values < 0.002), especially during days 3 and 4 (Figure 7). These included genes involved in some major transport systems, such as the ABC-type efflux porter complex (up to 11 fold-change, TukeyHSD adj. *p*-values < 0.05), the major facilitator superfamily (MFS; up to 6.4 fold-change, TukeyHSD adj. *p*-values < 0.04), the TonB-dependent receptor-like (up to 4.5 fold-change, TukeyHSD adj. *p*-values < 0.045), or the Type II/III secretion system (up to 4.2 fold-change, TukeyHSD adj. *p*-values < 0.04).

#### Genes Involved in Cell Motility

Sequences assigned to flagellum-dependent motility (GO:0071973), bacterial flagellum (GO:0009288), signaling receptor activity (GO:0038023), signal transduction (GO:0007165), and response to chemicals/chemotaxis (GO:0042221) were overall overrepresented in the tDOM treatments (up to 2.8 fold-change, TukeyHSD adj. *p*-values < 0.045), principally at days 3 and 4 (Figure 7). A number of genes encoding for a variety of flagellar proteins were found to be markedly overrepresented (e.g., *Flag_FliH*, 14 fold-change in DOC_70_-Hg^+^ at day 3, TukeyHSD adj. *p*-value < 0.0001). These genes represented different protein components involved in the bacterial-type flagellum-dependent cell motility and chemotaxis, such as the flagellar motor switch (e.g., *Flg_Motor_Flig* and *FliN_T3SS_HrcQb*) or the flagellar basal body (e.g., *FlgMring_FliF*, *Flagell_FlgL*, and *Flag_FlgI*).

### *hgcAB* and *mer* Gene Clusters

Genes present in the *mer* gene cluster were detected at very low abundances, representing 0.004% of the total sequences assigned to functional genes (Table 2). Five genes from the *mer* operon—*MerB, MerC, MerE, MerR*, *and MerT*—were detected in all treatments, including the unexposed control, with no significant differences between treatments. The *hgcAB* gene pair was not detected in the unexposed control, and only an *hgcA*-like gene belonging to the CdhD family (CO dehydrogenase/acetyl-CoA synthase complex beta subunit) was detected in the three Hg treatments, although statistical differences with the control were not significant.

**Table 2:** Abundance (normalized read counts) of genes involved in the biochemical cycle of Hg.

Genes	Control	Hg^+^	DOC_40_-Hg^+^	DOC_70_-Hg^+^	Control	Hg^+^	DOC_40_-Hg^+^	DOC_70_-Hg^+^	Control	Hg^+^	DOC_40_-Hg^+^	DOC_70_-Hg^+^	Control	Hg^+^	DOC_40_-Hg^+^	DOC_70_-Hg^+^
	Day 3				Day 4				Day 8				Day 12			
*merB*	**2** ± 1	**4** ± 1	**2** ± 1	**2** ± 1	**2** ± 3	**2** ± 0	**2** ± 1	**2** ± 1	**2** ± 1	**3** ± 2	**2** ± 0	**2** ± 1	**2** ± 1	**3** ± 1	**2** ± 1	**2** ± 1
*merC*	**5** ± 1	**6** ± 2	**6** ± 2	**5** ± 0	**7** ± 1	**9** ± 1	**7** ± 3	**12** ± 1	**10** ± 1	**12** ± 1	**13** ± 6	**14** ± 4	**13** ± 3	**14** ± 3	**14** ± 5	**12** ± 2
*merE*	**0** ± 0	**0** ± 0	**1** ± 1	**1** ± 1	**0** ± 0	**0** ± 0	**0** ± 0	**0** ± 0	**0** ± 0	**0** ± 0	**0** ± 0	**0** ± 0	**0** ± 0	**0** ± 0	**1** ± 1	**0** ± 0
*merR*	**1** ± 1	**2** ± 1	**1** ± 1	**2** ± 1	**2** ± 1	**0** ± 1	**1** ± 0	**0** ± 0	**2** ± 1	**1** ± 1	**1** ± 1	**1** ± 1	**3** ± 2	**2** ± 2	**1** ± 1	**1** ± 1
*merT*	**1** ± 2	**2** ± 0	**1** ± 1	**2** ± 1	**3** ± 3	**3** ± 0	**2** ± 1	**2** ± 1	**3** ± 1	**2** ± 0	**2** ± 1	**1** ± 1	**2** ± 1	**2** ± 2	**3** ± 2	**2** ± 2

Average values (bold) and standard deviation are shown.

## Discussion

### MeHg Formation in Hg(II) Amended Surface Coastal Waters

A number of studies have reported MeHg in the oxic layers of different aquatic systems (Monperrus et al., 2007; Lehnherr et al., 2011; Paranjape and Hall, 2017), which has led to the hypothesis that MeHg can also be formed in oxic waters. In the present study, the concentration of MeHg in experimental microcosms containing oxic surface waters collected from the pelagic zone of the Bothnian Sea was found to increase within 27 h after the addition of Hg(II) at 250 pM and under ambient DOC concentration (i.e., treatment Hg^+^). While methylation of mercury may occur to some extent through chemical processes (e.g., ultraviolet radiation and reaction with humic and fulvic acids), microbial metabolism is the main source of MeHg in aquatic environments (Celo et al., 2006). The magnitude of MeHg production observed in the present study is unlikely to be solely attributed to chemical processes, but rather, it is reasonable to consider that bacterial activity played a central role. This is supported by the fact that a strong positive correlation between bacterial production and MeHg concentration was observed during the first 3 days of incubation.

The increase in MeHg and bacterial production was not accompanied by alterations in bacterial community structure, diversity, or gene pool. It is possible that rapid, transient shifts in gene expression and enzymatic activity did occur while maintaining a similar genomic toolbox (Yu and Zhang, 2012; Pandit et al., 2018). The present study does not provide direct information from gene expression or enzymatic activity, but the shotgun metagenomics approach employed offers insights into the functional potential of the bacterial communities. High degree of functional redundancy is an essential property of natural bacterial communities that confers high levels of resistance and resilience under external environmental disturbance, especially if the disturbance takes place over brief periods or at a relatively low magnitude (Allison and Martiny, 2008; Bissett et al., 2013). The concept of resistance has been defined ecologically as the capacity of a community to withstand structural changes induced by environmental disturbance. Within the complexity of natural bacterial assemblages, different members of the community can perform similar ecological functions (i.e., functional redundancy), resulting in significant changes in metabolic performance with small changes in community structure (Allison and Martiny, 2008; Bissett et al., 2013). In these situations, function, diversity, and community composition may be decoupled. Such processes could explain the similar patterns in community structure observed in the present study between the treatment with the addition of Hg(II) and the unexposed control while showing differences in MeHg concentration that suggest higher microbial Hg methylation activity. In fact, genes encoding for enzymes suggested to participate in metabolic pathways involved in the methylation of Hg were found to be present in the bacterial communities from all treatments, such as methylene-THF dehydrogenase (*HF_DH/CycHdrlase*), 5,10-Methylene-THF-reductase (*MTHF_reductase_bac*), serine hydroxymethyltransferase (*Ser_HO-MeTrfase*), and 5-methyltetrahydrofolate-homocysteine S-methyltransferase (*MetH*) (Qian et al., 2016). With the exception of *Ser_HO-MeTrfase*, these genes were not differentially abundant between the treatments; nonetheless, they were part of the community gene pool and therefore may have played an important role in the increase of MeHg observed by upregulation of gene expression and enzymatic activity. Further studies focused on gene expression and/or enzymatic activity are required to address this aspect.

### Low Occurrence of *hgcAB* and *mer* Gene Clusters

Genes traditionally described to be involved in Hg methylation and cycling (e.g., *hgcAB* and *mer* genes) were scarcely detected in this study and did not show significantly different abundances when Hg(II) was added. However, apart from the genes encoding for mercury-specific transporters (*MerT* and *MerC*), other metal transporters have been shown to be involved in the uptake of Hg(II), for example, transporters for metal ions such as Zn(II), Cu(II), and Mg(II) (Regier et al., 2013; Schaefer et al., 2014), whose genes were also detected in this study (e.g., *ZnuA*, *MgTranspt_CorA/ZnTranspt_ZntB*, and *Mg/Co-transport_prot_CorA*). In these cases, the uptake of Hg(II) responds to competitive binding dynamics where some transporters show a higher affinity for specific metals, such as Zn or Cu (Mastropasqua et al., 2017). Thus, the addition of such metals at high concentrations has been shown to decrease the uptake of Hg(II) and consequently Hg methylation (Haas and Franz, 2009; Szczuka et al., 2015). Likewise, increasing concentrations of Hg(II) may conceivably lead to increased Hg methylation as a result of higher internalization of Hg(II) by these transporters. In addition, the ABC transporters (ATP-binding cassette transporters), for which a number of encoding genes showed increased abundances when tDOM was added, have been reported to be involved in the active uptake of different metal ions, including Hg (Qian et al., 2016).

Furthermore, Hg methylators widely reported to carry *hgcAB* genes, such as sulfate/iron-reducing bacteria and methanogens (Gilmour et al., 2013; Podar et al., 2015), were not detected in this study. *hgcAB* gene pair encodes for a corrinoid protein and an iron–sulfur cluster protein. We detected the presence of a *hgcA*-like gene belonging to the CdhD family (CO dehydrogenase/acetyl-CoA synthase complex beta subunit), and sequences assigned to the iron–sulfur cluster assembly were underrepresented when tDOM was added compared to the unexposed bacterial communities. In line with our results, Malcolm et al. (2010) found no evidence for anaerobic methylators in oxygen deficient oceanic waters where Hg methylation was detected, concluding that anaerobic bacteria do not play a substantial role in the methylation of Hg in the open ocean. Other studies have also reported Hg methylation in oxic marine waters (e.g., Monperrus et al., 2007; Lehnherr et al., 2011), where *hgcAB* genes are generally rarely present (Podar et al., 2015).

### tDOM Addition Enhances MeHg Formation

Dissolved organic matter has been extensively described to promote Hg methylation as well as transport and bioaccumulation of MeHg through different processes, such as enhancing microbial metabolism (Hall et al., 2004; Paranjape and Hall, 2017), changes in the physiology/permeability of bacterial cell membrane (Campbell et al., 1997; Vigneault et al., 2000), and its influence as complexing agent on Hg(II) speciation (Hsu-Kim et al., 2013; Jiang et al., 2018). In the present study, the addition of tDOM in the form of a soil extract led to increased MeHg concentrations in the seawater at the early stages of the experiment compared to the unexposed control. This increase was greatest by day 4 when DOC was raised by ~70% (*circa* 6.8 mg L^−1^) from natural levels, while increasing DOC concentration by ~40% (*circa* 5.6 mg L^−1^) did not result in a significant overall increment of MeHg compared to natural seawater exposed to Hg(II) addition (i.e., treatment Hg^+^). A number of studies have reported positive correlations between concentrations of MeHg and organic matter in surface and intermediate marine waters (Cossa et al., 2009; Sunderland et al., 2009; Kirk et al., 2012), which have been attributed to the microbial decomposition of settling particles where micro-anoxic environments are created, allowing anaerobic Hg methylators (e.g., iron–sulfur reducing bacteria and methanogens) to be active (Mason et al., 2012; Ortiz et al., 2015). However, in the present study, the seawater was filtered through 0.7 μm and the tDOM extract through 0.2 μm, so large particles were eliminated from the experimental water. In addition, we did not observe sedimentation/flocculation of particles onto the bottom of the microcosms over the experiment, although the formation of small particles during the experimental phase cannot be completely excluded. Therefore, it appears reasonable to assume that, in the aerobic environment where MeHg concentration significantly increased from ambient levels, bacterial communities were responsible for MeHg production.

In addition, recent studies have shown connections between Hg availability/methylation and increasing concentration of DOM and DOC. In an experiment using *Escherichia coli* as a bacterial model, Chiasson-Gould et al. (2014) observed that Hg(II) availability followed a bell-shaped pattern with increasing concentrations of DOM, where internalization of HgII steadily increased up to a threshold of ~8.6 mg L^−1^ DOC, after which Hg(II) availability decreased when exposed to higher DOC concentrations. French et al. (2014) reported similar results in a study where DOC was found to increasingly promote MeHg bioaccumulation in aquatic invertebrates from Arctic lakes with a DOC gradient up to ~8.5 mg C L^−1^, with higher concentrations affecting negatively the bioavailability of Hg and MeHg. In line with these findings, we observed increasing MeHg production when DOC was raised from ambient levels (4.05 mg L^−1^) up to 6.8 mg L^−1^, which provides evidence for similar DOM-MeHg dynamics in brackish waters.

### Rapid Responses in Bacterial Biochemical Activity and Methylmercury Formation Following tDOM Amendment

An increment of MeHg was observed to occur within the first 27 h after the addition of Hg(II). Overall, our results from bacterial production, diversity, community structure, and changes in gene abundances point to a rapid response of bacterial communities following the addition of tDOM and Hg(II). The transient alterations in bacterial production, community structure, and gene pool observed during the first days of the experiment were likely the result of a boosting effect of tDOM on bacterial activity, where bioavailable DOC may have been consumed during the initial stages of the experiment, after which these bacterial parameters subsequently returned toward values similar to those observed in the unexposed control (Dinasquet et al., 2013; Attermeyer et al., 2014). In addition, an increased bacterial activity boosted by tDOM addition may have led to higher Hg methylation by the resident bacterial communities (Hall et al., 2004), as suggested by the strong correlations found between bacterial production and MeHg concentration over the first days after the addition of Hg(II). In support of these observations, we detected increased bacterial production and higher abundances of genes related to enzymatic activity, redox processes associated with energy generation and cell motility when tDOM was added. Furthermore, most of the differences found in the abundance of a variety of functional genes occurred within the first 27 h after Hg(II) addition, particularly when tDOM was added, after which their abundances tended to converge with the values found in the unexposed bacterial communities. These observations support the idea of Hg methylation being a side effect of increased bacterial production, rather than being purely a detoxification mechanism (Paranjape and Hall, 2017).

Some studies suggest the effects of DOM on Hg methylation due to its impact on cell physiology and Hg(II) speciation, thus influencing Hg bioavailability (Vigneault et al., 2000; Gorski et al., 2008; Chiasson-Gould et al., 2014). Before Hg becomes complexed with different molecules present in the DOM pool (i.e., non-equilibrium conditions), the presence of DOM can facilitate the cell uptake of freshly deposited Hg by altering the bacterial membrane properties. However, this Hg-DOM complexation can occur within a few hours (~24 h) after Hg or DOM has entered the aquatic system, after which the bioavailability of Hg for bacterial uptake is reduced. In the present study, bacterial communities exposed to increased levels of tDOM and Hg(II) showed higher abundances of genes related to transmembrane transport activity, which suggests changes in membrane permeability that may result in an increased transport of ions and molecules such as Hg(II) and MeHg.

## Conclusion

In the present study, we addressed three key aspects regarding MeHg formation still under debate: (1) MeHg formation in oxic pelagic waters, (2) enhanced MeHg formation under increased tDOM and bacterial activity levels, and (3) rapid response of bacterial biochemical activity leading to MeHg formation within few hours after the input of Hg(II) to oxic DOM-rich waters. Our results show that methylation of Hg can occur in oxic brackish coastal waters, this being a fast process that can take place before Hg-DOM equilibrium has been reached, which is coupled to high bacterial biochemical activity induced by increased tDOM concentration. The enhanced Hg methylation activity was not linked to any substantial changes in bacterial community composition or gene pool. These findings underline relevant ecological implications in a changing global climate where increased terrestrial runoff in the northern hemisphere is expected to exacerbate the entrance of tDOM and environmental pollutants (including Hg) into coastal waters.

Shotgun metagenomics offers a wide perspective of the taxonomic composition and functional potential of bacterial communities, allowing the detection of overall responses and functioning shifts under fluctuating environmental factors. In order to further improve our insight into the metabolic response of bacterioplankton communities under increased levels of Hg and tDOM, future studies focused on gene expression (e.g., metatranscriptomics or qPCR) and enzymatic activity are required, which will contribute to disentangling the metabolic mechanisms involved in the methylation of Hg in oxic aquatic environments and its interactions with organic matter.

## Data Availability Statement

The data presented in the study are deposited in the NCBI BioProject repository, accession number PRJNA777782.

## Author Contributions

JR, EB, AA, OR, and SB designed the microcosm experiment. AS and EB carried out the Hg and MeHg analyses and preparation of the soil extract. JR and SB carried out the sampling over the experiment. SB carried out BP and chlorophyll-a analyses. JR, OR, and ST designed the molecular work. JR performed all molecular analyses, all bioinformatics analyses, and all statistical analyses and conducted the manuscript writing and editing. OR, ST, AA, EB, and SB provided support and supervision during manuscript drafting. All authors contributed to the article and approved the submitted version.

## Funding

This work was financed by Maj and Tor Nessling Foundation (project nos. 201500403, 201600062, and 201700320), the Swedish marine strategic research program EcoChange (Swedish research council FORMAS), and the Swedish Environmental Protection Agency (GD-2021-0003).

## Conflict of Interest

The authors declare that the research was conducted in the absence of any commercial or financial relationships that could be construed as a potential conflict of interest.

## Publisher’s Note

All claims expressed in this article are solely those of the authors and do not necessarily represent those of their affiliated organizations, or those of the publisher, the editors and the reviewers. Any product that may be evaluated in this article, or claim that may be made by its manufacturer, is not guaranteed or endorsed by the publisher.

## Footnotes

^1^ www.scilifelab.se

^2^ http://www.ncbi.nlm.nih.gov/bioproject/777782
