# Supplementary material for: Inputs of Terrestrial Dissolved Organic Matter Enhance Bacterial Production and Methylmercury Formation in Oxic Coastal Water
Source: Front Microbiol. 2022 Jul 27;13:809166. doi: 10.3389/fmicb.2022.809166 (PMC9363918; doi:10.3389/fmicb.2022.809166)
Supplement: Supplementary file 1 [file Data_Sheet_1.PDF]

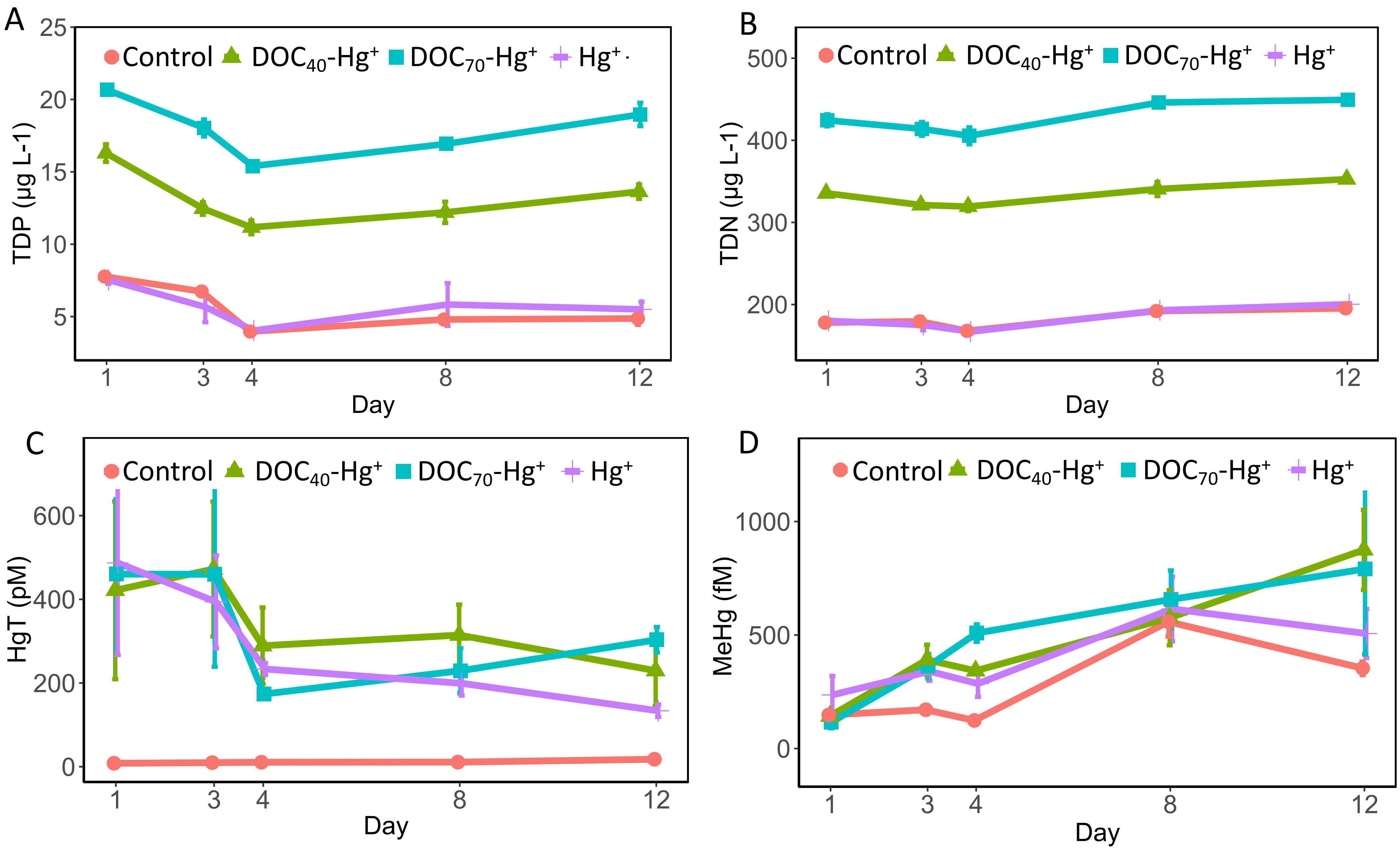

Figure S1. Concentration of total dissolved phosphorous (**A**), total dissolved nitrogen (**B**), total mercury (**C**), and methylmercury (**D**) in the different treatments throughout the experiment. Error bars represent the standard error (n = 3). Due to high variability of total mercury samples from days 1 and 3, these two time points were not further considered in this study.
