# Supplementary material for: Inputs of Terrestrial Dissolved Organic Matter Enhance Bacterial Production and Methylmercury Formation in Oxic Coastal Water
Source: Front Microbiol. 2022 Jul 27;13:809166. doi: 10.3389/fmicb.2022.809166 (PMC9363918; doi:10.3389/fmicb.2022.809166)
Supplement: Supplementary file 6 [file Data_Sheet_6.PDF]

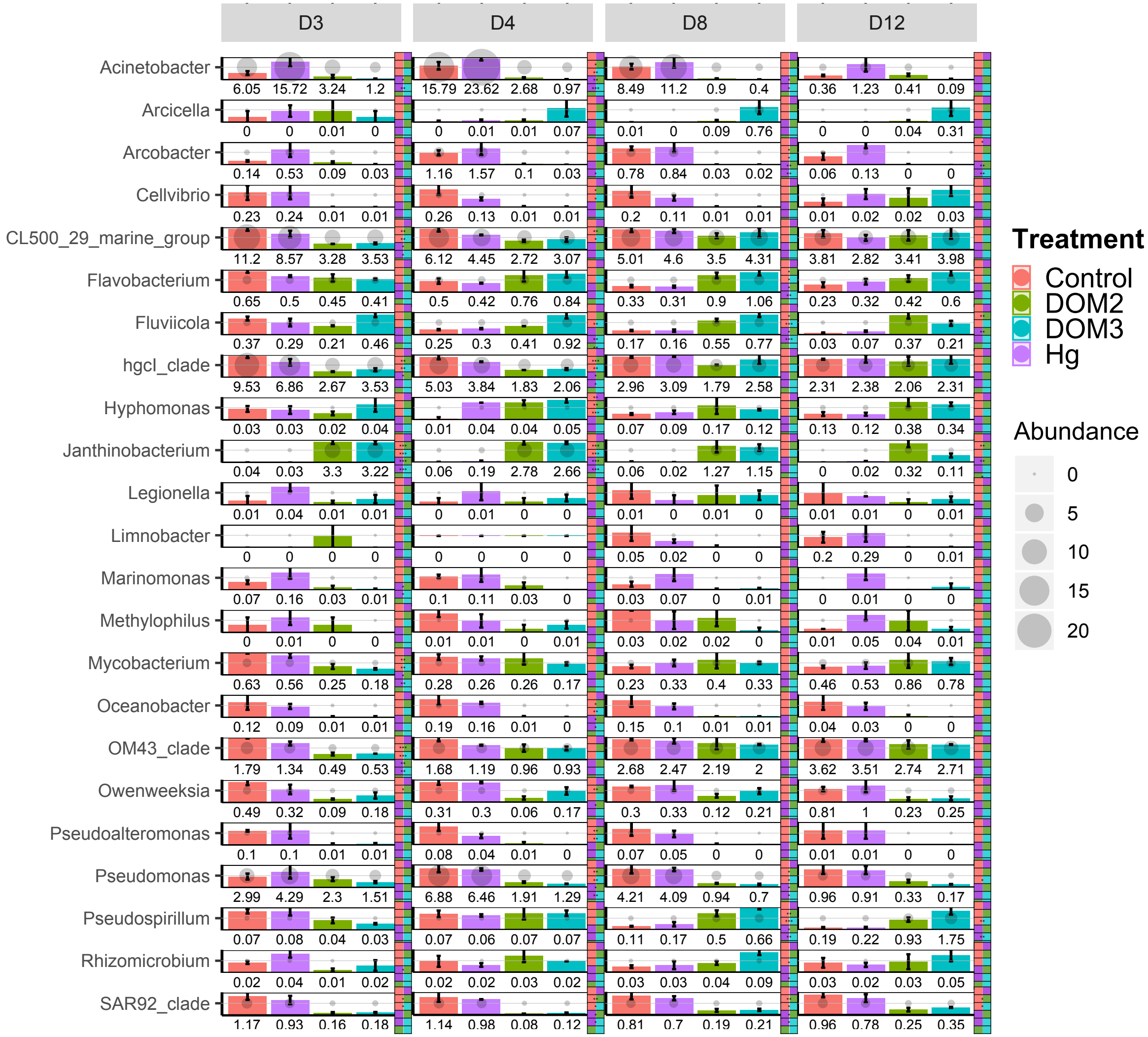

Figure S2E. Bar-balloon charts showing relative abundance (%) of bacterial genera throughout the experiment. Bars refer to intra-sample relative abundances, while balloons indicate inter-sample relative abundances. The significance of pair-wise comparisons (TukeyHSD) between the treatments is shown as asterisks (signif. codes: $p < 0.001$ ***; $p < 0.01$ *; $p < 0.05$ *).
