# Supplementary material for: Inputs of Terrestrial Dissolved Organic Matter Enhance Bacterial Production and Methylmercury Formation in Oxic Coastal Water
Source: Front Microbiol. 2022 Jul 27;13:809166. doi: 10.3389/fmicb.2022.809166 (PMC9363918; doi:10.3389/fmicb.2022.809166)
Supplement: Supplementary file 8 [file Data_Sheet_8.PDF]

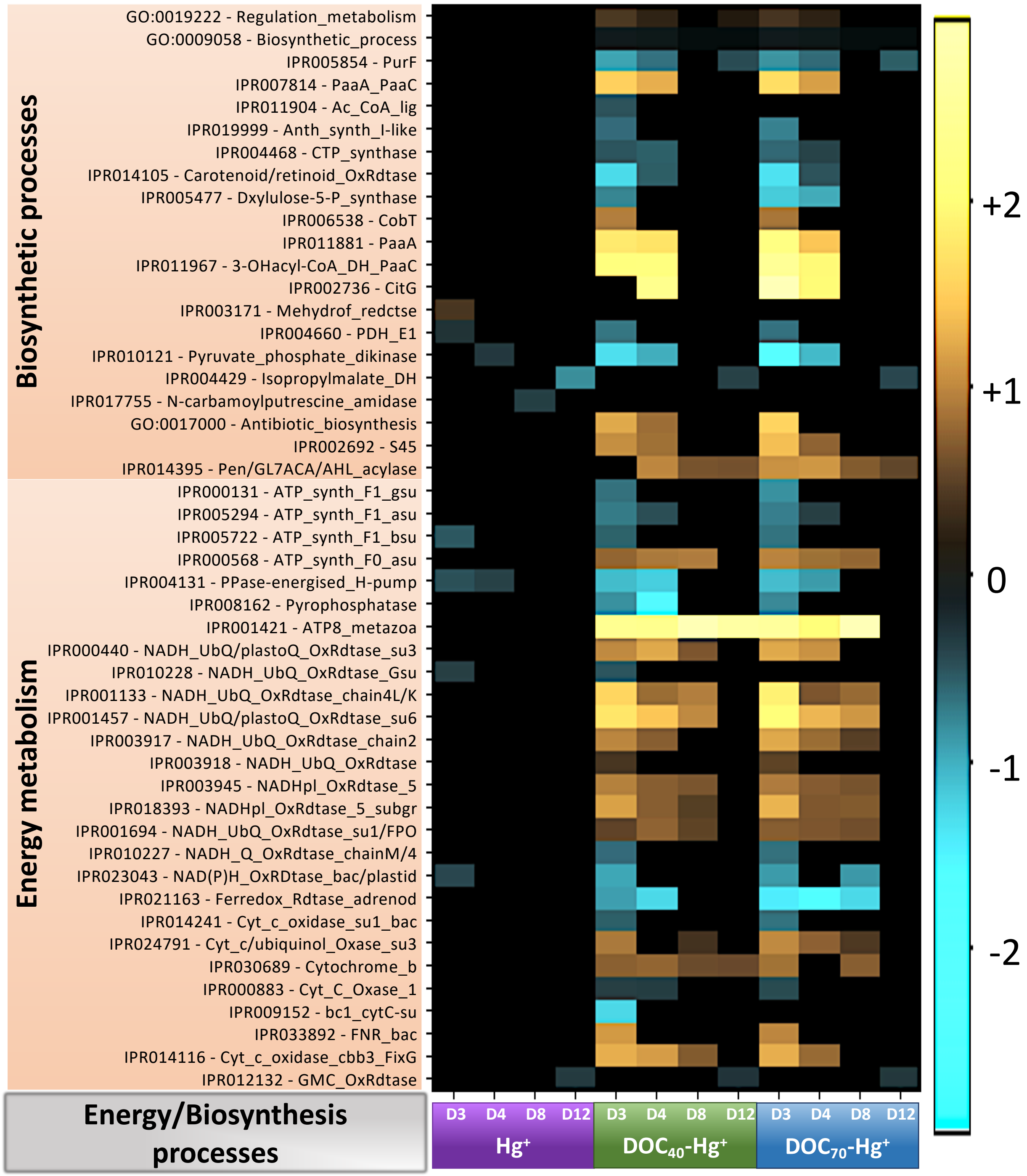

Figure S3B. Heatmaps showing fold-change abundance (Log_2 fold-change) of functional genes involved in biosynthetic/catabolic processes and in the generation of precursor metabolites and energy from treatments Hg^+, DOC_{40}-Hg^+ and DOC_{70}-Hg^+ compared to the unexposed control. Warm colours (yellow) indicate overrepresentation (i.e., higher abundances), colder colours indicate underrepresentation, and black indicates non-significance ($p > 0.05$).
