# Supplementary material for: Inputs of Terrestrial Dissolved Organic Matter Enhance Bacterial Production and Methylmercury Formation in Oxic Coastal Water
Source: Front Microbiol. 2022 Jul 27;13:809166. doi: 10.3389/fmicb.2022.809166 (PMC9363918; doi:10.3389/fmicb.2022.809166)
Supplement: Supplementary file 11 [file Data_Sheet_11.PDF]

Appendix 1

The following sections describe differential abundance of a variety of functional genes included in various GO categories.

DNA metabolic processes (**Figure S3A**). Sequences assigned to genes involved in DNA metabolic processes (GO:0006259) and DNA conformation change (GO:0071103) were overall slightly underrepresented at days 3 and 4 in treatments with tDOM addition compared to the unexposed control (up to 1.3 fold-change, TukeyHSD adj. p-values < 0.03). Among them, we found genes encoding for different types of DNA topoisomerases (*Topo_IA*, *Topo_IIA*, *Topo_IIA_B*) and DNA gyrases (*GyrA*, *GyrB*). On the other hand, genes involved in DNA transcription and repair processes were slightly overrepresented at days 3 and 4 in tDOM treatments (up to 1.39 fold-change, TukeyHSD adj. p-values < 0.02), although some genes encoding for DNA polymerases were found to be underrepresented (e.g. *PolC_alpha*, *DNA_pol_III_sug/sutau* and *DNA_polymerase_1*). Two genes encoding for transcription regulators (*Tscrpt_reg_AsnC-typ* and *Tscrpt_reg_HTH_AraC-type*) were found in higher abundance (1.3 - 1.66 fold-change, TukeyHSD adj. p-values < 0.05) in tDOM treatments at days 3 and 4, whereas *Term_rho* (transcription termination factor Rho) was significantly underrepresented compared to the control (up to 2.6 fold-change, TukeyHSD adj. p-value < 0.0001). Genes related to regulation of DNA repair (GO:0006282) were overall significantly overrepresented at days 4 and 12 in both tDOM treatments, although genes encoding for Rec proteins (*DNA_recomb/repair_RecA* and *DNA_recomb/repair_RecN*) and UvrABC system (*UvrA* and *UvrB*) were found in lower abundances (up to 1.8 fold-change, TukeyHSD adj. p-values < 0.05). Furthermore, *Fan1-like* (DNA repair nuclease) was found to be underrepresented in the three HgII treatments at day 4, particularly in Hg^+ treatment (2.3 fold-change, TukeyHSD adj. p-value = 0.006).

Translation (**Figure S3A**). Sequences assigned to genes involved in translation (GO:0006412) and RNA metabolic processes (GO:0016070) were found to be slightly underrepresented in the tDOM treatments during days 3 and 4. Three genes encoding for tRNA synthetases (*aa-tRNA-synth_Ic*, *Met-tRNA_synth_2* and *Met-tRNA_synth*) were found in significantly lower abundances compared to the control at day 3 in both tDOM treatments (up to 2.4 fold-change, TukeyHSD adj. p-values < 0.04). The ribosome hibernation promoting factor *RHF/RaiA* was found in significantly higher abundance in both tDOM treatments at day 12 (1.5 fold-change, TukeyHSD adj. p-values < 0.03).

Nucleotide metabolic processes (**Figure S3A**). Significant differences were consistently detected during days 3 and 4, with greater fold-changes observed in the treatments with tDOM addition. These genes were involved in metabolic pathways of purines and pyrimidines, particularly uridine monophosphate (UMP), the precursor of all pyrimidine nucleotides, for which genes such as *DHOD_1B*, *OMPdecase* and *Uridine_phosphorylase* were found in significantly lower abundances in the three Hg(II) treatments, with greater differences (up to 6.2 fold-change; TukeyHSD adj. p-values < 0.03) observed in the treatments with tDOM addition.

Energy/Redox activity (**Figure S3B**). Although genes related to the generation of precursor metabolites and energy (GO: 0006091) did not show overall significant differences between treatments, genes involved in proton transmembrane transport (e.g., *ATP_synth_F1_bsu* and *PPase-energised_H-pump*) and oxidation-reduction processes (e.g., *NADH_UbQ_OxRdtase_Gsu*, *NAD(P)H_OxRDtase_bac/plastid* and *GMC_OxRdtase*) were found to be significantly underrepresented in all Hg^+ treatments. These differences were generally observed at days 3 and 4, and enhanced in the treatments with tDOM addition (e.g., 2.3 fold-change in DOC_{40}-Hg^+ at day 4, TukeyHSD adj. p-values < 0.0001). In contrast, a

number of genes encoding for NADH:ubiquinone oxidoreductases (e.g., *NADH_UbQ_OxRdtase_chain4L/K*, *NADH_UbQ/plastoQ_OxRdtase_su6*, and *NADHpl_OxRdtase_5*) were found in significantly higher abundances in the tDOM treatments throughout days 3-8. Likewise, genes involved in the generation of electrochemical potential coupled to ATP synthesis were found to be underrepresented (e.g., *Ferredox_Rdtase_adrenod*, 3 fold-change in DOC_{70}-Hg^+ at day 4, TukeyHSD adj. p-value = 0.002) as well as overrepresented (e.g., *Cyt_c_oxidase_cbb3_FixG*, 2.4 fold-change in DOC_{40}-Hg^+ at day 3, TukeyHSD adj. p-value = 0.003) particularly in the tDOM treatments.

Biosynthetic/catabolic processes (**Figure S3B**). Overall, genes involved in biosynthetic processes (GO:0009058) were slightly (yet significantly) underrepresented in both tDOM treatments compared to the control. A number of genes involved in the acetyl-CoA pathway via phenylacetate catabolic processes (i.e., *Paa* genes) were significantly more abundant in tDOM treatments at days 3 and 4, such as *PaaA_PaaC* (up to 3 fold-change, TukeyHSD adj. p-values < 0.03), *PaaA* (up to 4.3 fold-change, TukeyHSD adj. p-values < 0.05), and *3-OHacyl-CoA_DH_PaaC* (up to 5.3 fold-change, TukeyHSD adj. p-values < 0.01). Other genes involved in the acetyl-CoA pathway via pyruvate metabolic processes were underrepresented in all Hg treatments at different time points, e.g. *PDH_E1* (1.6 fold-change at day 3 in DOC_{70}-Hg^+, TukeyHSD adj. p-value < 0.001) and *Pyruvate_phosphate_dikinase* (4.6 fold-change at day 3 in DOC_{70}-Hg^+, TukeyHSD adj. p-value < 0.001). *Ser_HO-MeTrfase* (also involved in the acetyl-CoA pathway) was underrepresented in both tDOM treatments at day 3 and 4 (up to 1.6 fold-change, TukeyHSD adj. p-values < 0.02). Furthermore, sequences assigned to genes involved in antibiotic biosynthesis (GO:0017000) were significantly more abundant in tDOM treatments particularly during days 3 and 4 (up to 3 fold-change, TukeyHSD adj. p-values < 0.03).

Fe/S metabolic processes (**Figure S3C**). A number of genes involved in Fe/S metabolic processes were found to be underrepresented in the three Hg treatments compared to the control. Sequences assigned to genes related to iron-sulphur (Fe/S) cluster assembly (GO: 0016226) were overall at lower abundances in both tDOM treatments (up to 2 fold-change, TukeyHSD adj. p-values < 0.04), particularly at days 3 and 4. These genes encode for important cofactors involved in diverse metabolic processes such as redox catalysis, regulation of gene expression and mobilisation of Fe and S atoms from storage sources. For instance, two genes from the *suf* operon (*SUF_FeS_clus_asmbl_SufBD* and *IPR010231 - SUF_FeS_clus_asmbl_SufB*), which is involved in sulphur assimilation (SUF system), were found to be significantly underrepresented mainly at days 3 and 4 (up to 2.5 fold-change, TukeyHSD adj. p-values < 0.02). Furthermore, the gene encoding for the iron-sulphur cluster insertion protein ErpA (*FeS_cluster_insertion_RrpA*) was underrepresented by 1.6 - 1.8 fold-change in the three Hg treatments at day 12 (TukeyHSD adj. p-values < 0.01).

Nitrogen metabolic processes (**Figure S3C**). Sequences assigned to genes involved in the metabolism of nitrogen compounds (GO:0006807) were overall mildly underrepresented in the tDOM treatments. Despite the overall non-significant differences between Hg^+ treatment and the unexposed control, several genes involved in the assimilation of ammonia and glutamate biosynthesis (*Glut_synth_ssu1*, *Glut_synth_ssu2*, *Gln_synth_I* and *Glu_DH*) were found in significantly lower abundances in all Hg treatments, showing greater differences in the tDOM treatments (up to 2.3 fold-change; TukeyHSD adj. p-values < 0.05). Furthermore, two genes involved in the metabolism of ammonium (*Cyt_c552* and *Ammonium_transptr_marine*) were found to be significantly underrepresented during days 3 and 4 in the tDOM treatments (up to 4 fold-change, TukeyHSD adj. p-values < 0.035). On the other hand, three genes involved in nitrate reductase activity (*NarG*, *NO3_Rdtase_bsu*, and *NapC/NirT/NrfH*) were more abundant

in the three Hg treatments during days 3 and 4 (up to 7.8 fold-change, TukeyHSD adj. p-values < 0.05).

Aminoacid metabolism (**Figure S3C**)**.** A number of genes encoding for aminoacyl-tRNA ligases, such as *Tyr-tRNA-ligase_bac_1*, *Ile-tRNA-ligase_type2*, *Leu-tRNA-ligase*, and *Valyl-tRNA_ligase*, were found to be underrepresented principally in the tDOM treatments (up to 3 fold-change, TukeyHSD adj. p-values < 0.045).

Enzymatic activity (**Figure 5**)**.** Sequences assigned to genes involved in **peptidase activity** (GO:0008233) and **proteolysis** (GO:0006508) were overall slightly overrepresented in all Hg treatments, with a number of genes encoding for peptidases (e.g., *Peptidase_M1*, *Peptidase_M3B*, *Tricorn_protease*, *Pept_S49_pIV*, etc.) showing significantly higher abundances at different time points compared to the control (up to 6.5 fold-change, TukeyHSD adj. p-values < 0.05). Genes involved in **isomerase** (GO:0016853), **ligase** (GO:0016874) and **transferase** (GO:0016740) activities were overall at significantly lower abundances in the tDOM treatments, particularly during days 3 and 4 (up to 1.22 fold-change, TukeyHSD adj. p-values < 0.04). In addition, sequences related to **phosphatase** (GO:0016791), **peroxidase** (GO:0004601) and **hydrolase** (GO:0016787) activities were overrepresented particularly at days 3 and 4 (up to 1.4 fold-change, TukeyHSD adj. p-values < 0.035). As a notable example, the gene encoding for the acid phosphatase *AcpA* (involved in the hydrolysis of a variety of substances, such as some peptides, glucose, ATP, NADP, etc.) was found to be conspicuously overrepresented in both tDOM treatments throughout days 3-8 (up to 23.3 fold-change, TukeyHSD adj. p-values < 0.02).

Cellular components (**Figure S3D**)**.** Sequences assigned to cellular components such as **ribosomes** (GO:0003735), **chromosome** (GO:0005694), **cytoplasm** (GO:0005737), and **intracellular compartment** (GO:0005622) were underrepresented in the tDOM treatments compared to the control (up to 1.8 fold-change, TukeyHSD adj. p-values < 0.004), principally at days 3 and 4. On the contrary, sequences related to the **extracellular region** (GO:0005576; outermost structure of the cell), **membrane** (GO:0016020; phospholipid bilayer along with all the proteins and protein complexes embedded in it an attached to it), **outer membrane** (GO:0019867; external membrane of Gram-negative bacteria), and **plasma membrane** (GO:0019867; phospholipid bilayer and associated proteins) were found to be overrepresented in the tDOM treatments (up to 1.54 fold-change, TukeyHSD adj. p-values < 0.05), principally at days 3 and 4. A total of 21 genes encoding for ribosomal proteins were found to be present in lower abundances in tDOM treatments compared to the control (up to 35 fold-change, TukeyHSD adj. p-values < 0.05). Some of these genes (e.g., *Ribosomal_L4/L1e*, *Ribosomal_L6_bac-type*, *Ribosomal_S14_Z*, and *Ribosomal_S17*) were significantly underrepresented in all Hg^+ treatments generally at days 3 and 4.

Transport (**Figure 6**)**.** Sequences assigned to genes involved in transport processes (GO:0006810; that is, directed movement of substances or cellular components into or out of a cell, or between cells), transporter activity (GO:0005215) and transmembrane transport (GO:0015238) were overall overrepresented in both tDOM treatments (up to 1.5 fold-change, TukeyHSD adj. p-values < 0.002), especially during days 3 and 4. These included genes involved in some major transport systems, such as the ABC-type efflux porter complex (up to 11 fold-change, TukeyHSD adj. p-values < 0.05), the major facilitator superfamily (MFS) (up to 6.4 fold-change, TukeyHSD adj. p-values < 0.04), the TonB-dependent receptor-like (up to 4.5 fold-change, TukeyHSD adj. p-values < 0.045), or the Type II/III secretion system (up to 4.2 fold-change, TukeyHSD adj. p-values < 0.04). Among them, we found genes involved in the transport of carbohydrates (e.g., *ABC_transpr_RbsA*, *Porin_LamB*, *Anaer_Dcu_memb_transpt* and *MFS_sugar_transport-like*), proteins (e.g., *T2SS/T3SS*,

T2SS_GspE and *T3SS_IM_R*), aminoacids (e.g., *ABC_ATPase_HisP-typ*, *GltJ/AatQ* and *MATE_fam*) and ions and small molecules (e.g., *Porin_Gram-ve*, *Porin_Neis* and *Na-dicarboxylate_symporter*). In addition, some of these genes were found to be overrepresented in all Hg treatments, such as *T2SS_protein-H* (protein transport), SsuB (anion transport), KdpA (K^+ transport), or Anaer_Dcu_memb_transpt (carbohydrate transport).

Cell motility (**Figure 6**)**.** Sequences assigned to **flagellum dependent motility** (GO:0071973), **bacterial flagellum** (GO:0009288), **signalling receptor activity** (GO:0038023), **signal transduction** (GO:0007165), and **response to chemicals/chemotaxis** (GO:0042221) were overall overrepresented in the tDOM treatments (up to 2.8 fold-change, TukeyHSD adj. p-values < 0.045), principally at days 3 and 4. A number of genes encoding for a variety of flagellar proteins were found to be markedly overrepresented (e.g., *Flag_FliH*, 14 fold-change in DOC_{70}-Hg^+ at day 3, TukeyHSD adj. p-value < 0.0001). These genes represented different protein components involved in the bacterial-type flagellum-dependent cell motility and chemotaxis, such as the flagellar motor switch (e.g., *Flg_Motor_Flig* and *FliN_T3SS_HrcQb*) or the flagellar basal body (e.g., *FlgMring_FliF*, *Flagell_FlgL* and *Flag_FlgI*).
