# Supplementary material for: Inputs of Terrestrial Dissolved Organic Matter Enhance Bacterial Production and Methylmercury Formation in Oxic Coastal Water
Source: Front Microbiol. 2022 Jul 27;13:809166. doi: 10.3389/fmicb.2022.809166 (PMC9363918; doi:10.3389/fmicb.2022.809166)
Supplement: Supplementary file 12 [file Data_Sheet_12.PDF]

Table S1. Concentration of total mercury (THg), methylmercury (MeHg), MeHg normalized against control, and MeHg/THg ratios in the different treatments over the experiment. The values correspond to average concentrations from replicates (n = 3), followed by the standard error.

	Treatments	Day				
		1	3	4	8	12
THg (pM)	Control	8.27 ± 0.12	9.73 ± 1.85	10.70 ± 2.57	10.93 ± 2.35	17.85 ± 5.57
	DOC40-Hg+	421.59 ± 212.41	472.33 ± 161.28	289.09 ± 91.45	314.51 ± 72.88	229.58 ± 85.24
	DOC70-Hg+	460.34 ± 178	459.81 ± 220.9	173.67 ± 11.35	229.37 ± 53.59	303.44 ± 30.39
	Hg+	486.94 ± 219.88	394.04 ± 110.79	233.45 ± 14.17	199.31 ± 29.2	133.68 ± 14.63
MeHg (fM)	Control	147.77 ± 12.6	170.86 ± 4.96	123.63 ± 10.54	558.60 ± 65.38	353.62 ± 29.55
	DOC40-Hg+	142.79 ± 13.86	391.04 ± 66.08	342.46 ± 13.26	575.99 ± 121.16	875.11 ± 176.35
	DOC70-Hg+	115.89 ± 26.4	362.73 ± 53.95	508.62 ± 38.45	656.76 ± 127.61	791.05 ± 376.13
	Hg+	236.17 ± 83.3	340.12 ± 41.65	285.70 ± 57.85	614.96 ± 142.71	506.46 ± 108.13
ΔMeHg (fM)	Control	–	–	–	–	–
	DOC40-Hg+	-4.98 ± 26.25	220.18 ± 69.6	218.84 ± 16.74	17.41 ± 147.57	521.48 ± 156.85
	DOC70-Hg+	-31.88 ± 30.76	191.88 ± 49.69	384.00 ± 37.68	98.18 ± 86.11	437.43 ± 372.78
	Hg+	88.4 ± 71.47	169.27 ± 37.97	162.07 ± 61.7	56.38 ± 79.29	152.83 ± 96.72
MeHg/THg ratio	Control	17.91 ± 1.76	18.95 ± 3.85	13.59 ± 4.54	55.6 ± 11.48	27.2 ± 11.9
	DOC40-Hg+	0.53 ± 0.23	1.15 ± 0.49	1.4 ± 0.34	1.85 ± 0.12	5.01 ± 2.01
	DOC70-Hg+	0.62 ± 0.48	1.1 ± 0.33	2.92 ± 0.03	2.98 ± 0.55	2.47 ± 0.98
	Hg+	0.67 ± 0.3	1.18 ± 0.57	1.22 ± 0.23	3.45 ± 1.35	4 ± 1.11
